# Maternal and Foetal Cellular Immune Responses in Dams Infected With High- and Low- Virulence Isolates of *Neospora caninum* at Mid-Gestation

**DOI:** 10.3389/fcimb.2021.684670

**Published:** 2021-06-22

**Authors:** Marta García-Sánchez, Laura Jiménez-Pelayo, Patricia Vázquez, Pilar Horcajo, Javier Regidor-Cerrillo, Alejandro Jiménez-Meléndez, Koldo Osoro, Luis Miguel Ortega-Mora, Esther Collantes-Fernández

**Affiliations:** ^1^ Saluvet, Animal Health Department, Complutense University of Madrid, Madrid, Spain; ^2^ Regional Service for Research and Agri-Food Development (SERIDA), Villaviciosa, Spain

**Keywords:** *Neospora caninum*, virulence, pregnant cattle, cellular immune responses, pathogenesis

## Abstract

Bovine neosporosis is currently considered one of the main causes of abortion in cattle worldwide and the outcome of the infection is, in part, determined by *Neospora caninum* isolate virulence. However, the dam and foetal immune responses associated with this factor are largely unknown. We used a model of bovine infection at day 110 of gestation to study the early infection dynamics (10- and 20-days post-infection, dpi) after experimental challenge with high- and low-virulence isolates of *N. caninum* (Nc-Spain7 and Nc-Spain1H, respectively). In the present work, dam peripheral cellular immune responses were monitored twice a week from -1 to 20 dpi. At different time points, IFN-γ and IL-4 production was investigated in stimulated dam blood and the percentage of monocytes, NK cells, B cells and T cells (CD4+, CD8+ and γδ) in peripheral blood mononuclear cells (PBMC) were determined by flow cytometry. In addition, maternal iliofemoral lymph nodes and foetal spleen and thymus were collected at 10 and 20 dpi for the study of the same cell subpopulations. Peripheral immune response dynamics were similar after the infection with both isolates, with a significant increase in the percentage of CD4+ T cells at 6 and 9 dpi in PBMC, coincident with the higher levels of IFN-γ and IL-4 release. However, the levels of IFN-γ were significantly higher and an increase in CD8+ T cells at 9, 13 and 20 dpi was observed in the dams infected with Nc-Spain7. Nc-Spain1H infection induced higher IL4 levels in stimulated blood and a higher CD4+/CD8+ ratio in PBMC. The analysis of the maternal iliofemoral lymph node showed a significant enhancement in the percentage of NK, CD4+ and CD8+ T cells for the animals infected with the highly virulent isolate and euthanized at 20 dpi. Regarding the foetal responses, the most remarkable result was an increase in the percentage of monocytes at 20 dpi in the spleen of foetuses from both infected groups, which suggests that foetuses were able to respond to *N. caninum* infection at mid gestation. This work provides insights into how isolate virulence affects the maternal and foetal immune responses generated against *N. caninum*, which may influence the course of infection.

## Introduction

*Neospora caninum* infection is a major cause of bovine abortion worldwide. Over the last few years, bovine neosporosis has been studied intensively, but the pathogenesis is complex and poorly understood. Previous studies have demonstrated the influence of timing of the infection during pregnancy (Benavides et al., 2014; Almería et al., 2017) and the *N. caninum* isolate (Rojo-Montejo et al., 2009; Goodswen et al., 2013; Regidor-Cerrillo et al., 2014; Jiménez-Pelayo et al., 2019) on the outcome of infection in cattle. Specifically, *in vivo* studies in pregnant cattle carried out by our group have shown a high percentage of abortion upon Nc-Spain7 infection with foetal death estimated to occur between 24 and 49 dpi (Caspe et al., 2012; Regidor-Cerrillo et al., 2014; Vázquez et al., 2019). In contrast no foetal death occurs in pregnant cattle experimentally infected with the Nc-Spain1H isolate (Rojo-Montejo et al., 2009). Recently, with the aim of exploring in more depth the mechanisms involved in the pathogenesis of abortion or vertical transmission, we used a pregnant bovine model of infection at mid-gestation, in which early infection dynamics (10- and 20-days post-infection, dpi) were compared after inoculation with the high-virulence Nc-Spain7 and the low-virulence Nc-Spain1H isolates. The results showed parasite presence and severe necrosis in placental tissues, as well as foetal mortality in Nc-Spain7-infected heifers. In contrast, Nc-Spain1H infection did not result in lesion development or foetal death (Jiménez-Pelayo et al., 2019). These results were associated with a differential pattern in the local immune response at the placental level, particularly in the early stages of infection. The low-virulence isolate Nc-Spain1H elicited an early and robust immune response characterized by a mixed Th1/Th2 profile, whereas a more predominantly pro-inflammatory Th1-based response was induced in Nc-Spain7-infected placentomes (Jiménez-Pelayo et al., 2020).

Cell-mediated immune (CMI) responses involving innate immune cells such as monocytes, natural killer cells, and adaptive immune cells including T and B lymphocyte subsets, as well as the production of cytokines such as IFN-γ and IL-4 are thought to be determinant factors for bovine neosporosis pathogenesis (Innes et al., 2002; Almeria et al., 2003; Rosbottom et al., 2007; Bartley et al., 2012; Bartley et al., 2013; Almería et al., 2017). The aim of this study was to determine how *N. caninum* isolate virulence may influence peripheral maternal and foetal cellular immune responses during the initial events of infection (10 and 20 dpi) at mid-gestation. This research may help to identify key immune components that could influence the outcome of infection.

## Materials and Methods

### Ethics Statement

All protocols involving animals were approved by the Animal Research Ethics (reference number PROAE 25/2016), following the proceedings described in Spanish and EU legislations (Law 32/2007, R.D. 53/2013, and Council Directive 2010/63/EU). All animals were handled in strict accordance with good clinical practices and all efforts were made to minimize suffering.

### Experimental Design and Sampling

The experimental design has been described in detail in a previous publication (Jiménez-Pelayo et al., 2019). Briefly, pregnant Asturian heifers (n=24) were randomly distributed into three experimental groups. At 110 days of gestation, animals were inoculated intravenously (IV) with phosphate buffered saline (PBS; G-Control, n=6) or 10^7^ tachyzoites of *N. caninum* Nc-Spain7 isolate (G-NcSpain7, n=9) or Nc-Spain1H isolate (G-NcSpain1H, n=9). Three animals from G-Control and four animals from G-NcSpain7 and G-NcSpain1H were culled at 10 days dpi. The remaining heifers from each group were culled at 20 dpi.

Blood samples were collected by coccygeal venipuncture into EDTA- and lithium heparin-coated tubes (Becton–Dickinson and Company, UK) for the flow cytometry and lymphoproliferation assays, described in sections 2.4 and 2.5 respectively. The time schedule for the samplings was as follows: -1, 2, 6 and 9 dpi for all heifers used in the experiment and culled at 10 or 20 dpi (n=24), and 13, 16 and 20 dpi only for those heifers culled at 20 dpi (n=13). Animals were euthanized by an IV overdose of embutramide and mebezodium iodide (T61; Intervet, Spain) after sedation by the intramuscular injection of xylazine (Rompun; Bayer, Germany). At necropsy, maternal iliofemoral lymph nodes, and foetal thymus and spleen were collected and maintained fresh in RPMI 1640 medium (Gibco, UK) until further assay. Two heifers (3581 and 7934) from G-NcSpain7 that were culled at 20 dpi presented foetal mortality (Jiménez-Pelayo et al., 2019).

### Parasite Inocula

*Neospora caninum* tachyzoites of Nc-Spain7 and Nc-Spain1H isolates were routinely maintained in a MARC-145 cell line culture as previously described (Regidor-Cerrillo et al., 2008). Tachyzoites were collected by cell scraping from three-day growth cultures, and at least 80% of the parasitophorous vacuoles were undisrupted. Parasites were counted in a Neubauer chamber by Trypan blue exclusion and resuspended in PBS, preparing the inocula at the required dose of 10^7^ tachyzoites in a final volume of 2 mL. Animals were infected before one hour of parasite harvest to minimize loss of viability.

### Peripheral Blood Stimulation Assay and Quantification of IFN-γ and IL-4 Release

A total of 500 mL of heparinized blood samples was cultured in triplicate within two hours of collection in 24-well plates (Thermo Fisher Scientific, USA) with 500 mL RPMI supplemented with 10% foetal bovine serum (Thermo Fisher Scientific, Waltham, USA) and 1% penicillin-streptomycin-amphotericin B mixture (Lonza, Belgium) referred to as complete medium (CM), as described previously (Vázquez et al., 2019). Each blood replicate was also supplemented with *N. caninum* soluble extract antigen obtained from Nc-Spain7 tachyzoites (Alvarez-García et al., 2003) at 5 μg/mL, concanavalin (ConA) (Sigma-Aldrich, Spain) at 5 μg/mL as a positive control, and PBS as negative control. After 24 h of incubation (37°C, 5% CO_2_), culture supernatants were collected by centrifugation of plates at 1000 × g for 10 min at 4°C and stored at −80 °C until laboratory analysis for the evaluation of IFN-γ and IL-4 release.

Cytokine release was measured with commercial bovine IFN-γ and bovine IL-4 ELISA kits (Mabtech AB, Nacka Strand, Sweden) following the manufacturer’s guidelines. The cytokine concentrations were calculated by interpolation from a standard curve produced with recombinant cytokines provided with the kits. The colour reaction was developed by the addition of 3,3’,5,5’-tetramethylbenzidine substrate (TMB, Sigma-Aldrich, Spain). Plates were read at 450 nm.

### Phenotypic Analysis of Immune Cells by Flow Cytometry

For the analysis of immune cell subpopulations from peripheral blood mononuclear cells (PBMC), a total of 20 mL of bovine peripheral blood collected in EDTA-coated tubes was mixed with 20 mL of PBS and centrifuged at 650 g for 30 min at room temperature (RT). PBMC were separated by gradient density centrifugation (800 g for 30 min at RT) on Histopaque 1077 (Sigma-Aldrich, USA).

For the analysis of immune cell subpopulations from the maternal iliofemoral lymph node and foetal thymus and spleen, the organs were mechanically disaggregated with Polytron^®^ PT 1600E (Kinematica AG, Lucerne, USA) in 20 mL of CM. The mixture was filtered through 100 μm nylon cell strainers followed by 40 μm nylon cell strainers (VWR, Radnor, PA, USA) and centrifuged at 350 g for 10 min at 4°C. The obtained pellet was resuspended in 5 mL PBS and mononuclear cells were separated by gradient density centrifugation on Histopaque 1077 as described above.

Mononuclear cells obtained from peripheral blood or organs were incubated with a sodium bicarbonate solution (0.01 M) for 5 min to lyse the erythrocytes followed by washing with cold PBS. Then, the cells were pelleted in a V-bottom 96-well plate at a density of 2 × 10^5^ cells/well and incubated with 50 μL of diluted antibody (1:100) or PBS for 30 min on ice and protected from light. After incubation, the samples were washed with PBS and fixed with BD Cellfix (BD Bioscience, USA).

The percentages of helper (CD4+), cytotoxic (CD8+) and γδ (WC1+) T cells, B lymphocytes (CD21+), NK cells (CD335+) and monocytes (CD14+) were analysed using flow cytometry after staining with antibodies specific for bovine leucocyte subpopulations (**Table 1**). The percentage of positive cells for each marker was determined using a Becton Dickinson FACSCalibur cytometer (BD Bioscience, USA), by calculating the proportion of the subpopulation reactive with each specific antibody in relation to the total mononuclear cell number.

**Table 1:** List of antibodies used for analysis of cell subsets in PBMC, ileofemoral lymph node, fetal thymus and spleen.

Antibody	Conjugate	Supplier	Species	Type	Clone	Reference
**CD14**	FITC	Bio-Rad Laboratories (Pleasanton, CA, USA)	Mouse anti-bovine	Monoclonal	CC-G33	MCA2678F
**CD4**	Alexa Fluor 647				CC8	MCA1653A647
**CD8**	RPE				CC63	MCA837PE
**CD335**	Alexa Fluor 488				AKS1	MCA2365A488
**WC1**	FITC				CC15	MCA838F
**CD21**	FITC		Mouse anti-human		LB21	MCA1195F

### Statistical Analysis

A repeated measures ANOVA test, followed by Tukey´s multiple comparisons test, was performed to compare cytokine levels in culture supernatants and the percentage of immune cell subsets in PBMC. Due to the differences in the number of animals before and after sacrifice at 10 dpi, days -1 to 9 dpi and 13 to 20 dpi were analysed separately. Dunnett´s multiple comparisons post-test was used to compare cell subsets in PBMC at each time point *versus* -1 dpi (preinfection day) for each group. A difference in the percentage of cell subsets in PBMC between infected and pre-infection values was considered valid only if no similar changes were seen in control animals. Differences in immune cell subsets in the thymus, spleen and maternal iliofemoral lymph nodes at 10 and 20 dpi were evaluated using the non-parametric Kruskal-Wallis followed by Dunn’s multiple range test to compare groups. Statistical significance for all analyses was established with P < 0.05. All statistical analyses were carried out using GraphPad Prism 7 v.7.03 (San Diego, CA, USA) software.

## Results

### Nc-Spain7 Induced Higher and Earlier IFN-γ Production, but Nc-Spain1H Infection Led to Higher IL-4 Levels in Stimulated Blood

**Figure 1:**
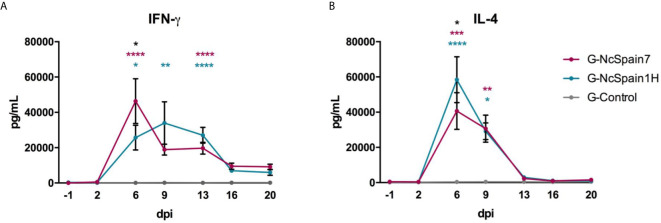
IFN-γ and IL-4 production after Nc-Spain7 and Nc-Spain1H infection. Concentrations of **(A)** IFN-γ and **(B)** IL-4 in response to *N. caninum* soluble extract antigen, in peripheral blood culture supernatants of heifers challenged with Nc-Spain7 (G-NcSpain7) and Nc-Spain1H (G-NcSpain1H) tachyzoites and the uninfected control group (G-Control). Each point represents the mean + SD at different sampling times. Asterisks indicate significant differences in G-NcSpain1H *versus* G-Control comparison (blue), G-NcSpain7 versus G-Control (red) and G-NcSpain1H *versus* G-NcSpain7 (black). ****P < 0.0001, ***P < 0.001, **P < 0.01, *P < 0.05.

The levels of IFN-γ and IL-4 were measured in stimulated blood by ELISA, and there was no antigen-specific IFN-γ or IL-4 secretion in the PBMC of the control animals (**Figure 1**). IFN-γ production increased faster and more markedly in G-NcSpain7, reaching the peak of production at 6 dpi (P<0.0001 *versus* G-Control), with differences observed at this time-point in relation to G-NcSpain1H (P < 0.05). For G-Nc-Spain1H the highest levels were observed at 9 dpi (P<0.01 *versus* G-Control), and the production was maintained over time (6-13 dpi) (**Figure 1A**). Then, IFN-γ decreased in both infected groups and significant differences were not found. Regarding IL-4, significantly higher levels were observed in the infected groups than in G-control at 6 and 9 dpi (P < 0.05-0.0001), but afterwards, the IL-4 levels returned to basal levels. The peak of production was observed at 6 dpi for both infected groups, being higher in the G-NcSpain1H (P < 0.05) (**Figure 1B**).

### Nc-Spain7 Triggered a Higher CD8+ Cells Percentage Whereas a Higher CD4+/CD8+ Ratio Was Induced by Nc-Spain1H Infection in PBMC

**Figure 2:**
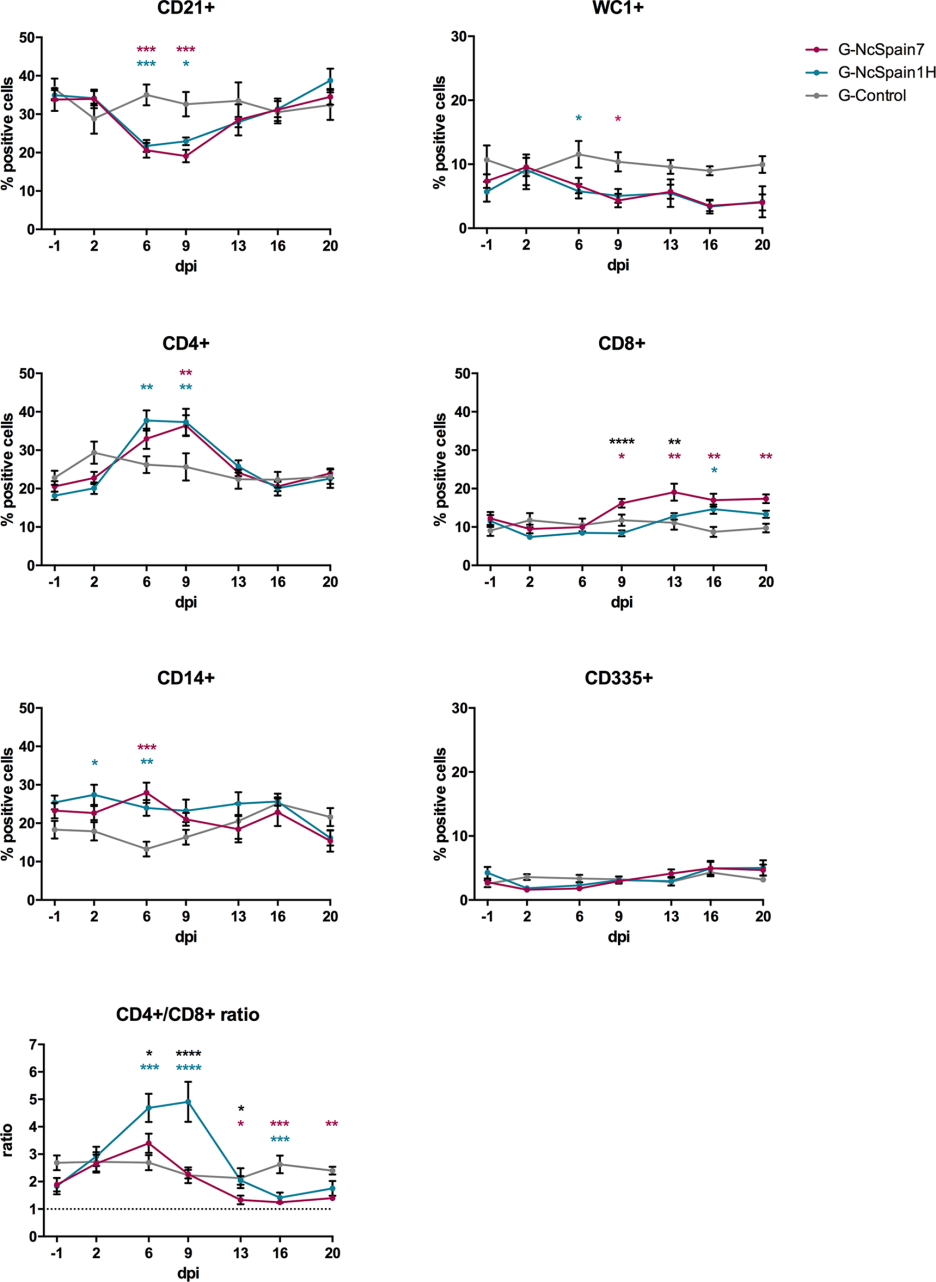
Relative percentage of immune cells in a PBMC population from Nc-Spain7- and Nc-Spain1H-infected heifers. Graphs indicate the relative percentage of cells positive for CD21, WC1, CD4, CD8, CD14 and CD335 surface markers, and CD4+/CD8+ ratio in PBMC obtained from uninfected heifers (G-Control) and heifers challenged with Nc-Spain7 (G-NcSpain7) and Nc-Spain1H (G-NcSpain1H) tachyzoites. The results are represented as the mean + SD. Asterisks indicate significant differences in G-NcSpain1H *versus* G-Control comparison (blue), G-NcSpain7 *versus* G-Control (red) and G-NcSpain1H *versus* G-NcSpain7 (black). ****P < 0.0001, ***P < 0.001, **P < 0.01, *P < 0.05.

The percentages of CD21+ (B cells), WC1+ (γδ-T cells), CD4+ (helper T cells), CD8+ (cytotoxic T cells), CD14+ (monocytes/macrophages) and CD335+ (NK) cells were studied in the population of PBMC from G-NcSpain7, G-NcSpain1H and G-Control by flow cytometry at different times post-infection (**Figure 2** and **Supplementary S1**). All values were within reference ranges, and there were no consistent changes over time in control animals (**Supplementary S1**). Significant differences with respect to the control group were observed in infected animals, mainly at 6 and 9 dpi. One of the most notable changes in PBMC subpopulations following *N. caninum* infection was an abrupt decrease in the relative percentage of the B lymphocyte subset (CD21+ cells) from 6 to 9 dpi *versus* uninfected animals (P < 0.05) and preinfection values (P < 0.001-0.0001). Then, relative percentages remained close to uninfected levels towards the end of the study. Similarly, in the infected groups, the relative percentages of γδ-T cells (WC1+) decreased gradually from 2 dpi, being significantly lower than those of non-infected animals at 6 dpi for G-NcSpain1H and 9 dpi for G-NcSpain7 (P < 0.05). In contrast, *N. caninum* infection induced a significant increase in the proportion of circulating CD4+, and CD8+ cells. In addition, the percentage of CD14+ cells was higher at 2 dpi in G-NcSpain1H (P < 0.05) and in both infected groups at 6 dpi (P < 0.01-0.001) *versus* G-control. However, CD14+ cell percentages were variable during the study period in both the control and infected animals, and we did not observe significant differences on day -1 *versus* 2 and 6 dpi in infected groups (**Supplementary S1**). The percentage of CD4+ cells also suffered an increase at 6 and 9 dpi for G-NcSpain1H (P < 0.01) and only at 9 dpi for G-NcSpain7 (P < 0.01) *versus* uninfected animals. When comparing with pre-infection values, the increase was statistically significant at 6 and 9 dpi for G-NcSpain7 (P < 0.001-0.0001) and at 6, 9 and 13 dpi for G-NcSpain1H (P < 0.05-0.0001). In general, the kinetics of immune cell subsets were essentially the same between G-NcSpain7 and G-NcSpain1H after inoculation. However, in G-NcSpain7, the CD8+ values increased at 9 dpi with respect to G-control (P < 0.05-0.01) and its own values before infection (P < 0.05-0.0001) remaining high until the end of the experiment, and the percentages were also higher *versus* G-NcSpain1H at 9 (P < 0.0001) and 13 dpi (P < 0.01). Nc-Spain1H induced only a modest CD8+ increase at 16 dpi (P < 0.05), which was only significant compared to the control group. Unlike the other cell subpopulations studied, there were no obvious changes in the relative percentage of the CD335+ (NK) subpopulation.

The ratio between helper (CD4+) and cytotoxic (CD8+) T cells was calculated to assess the immunoregulatory balance of T cells. In G-Control, the CD4+/CD8+ ratio was ∼2.5 during the experimental period. For G-NcSpain1H, the ratio significantly increased from 6 dpi (∼4.7) to 9 dpi (∼4.9) due to the increase in CD4+ T cells in circulating blood (G-NcSpain1H versus G-Control: P < 0.001-0.0001; G-NcSpain1H *versus* G-NcSpain7: P < 0.05-0.0001). From 13 dpi onwards, the CD4+/CD8+ ratio decreased following a similar profile to that observed for G-NcSpain7, which was lower than the value observed in the uninfected animals. The ratio was significantly lower for G-NcSpain7 from 13 to 20 dpi (P < 0.05-0.001) and for G-NcSpain1H only at 16 dpi (P < 0.001) due to the increase in CD8+ percentages in relation to G-Control.

### Nc-Spain7 Infection Induced Changes in Immune Cell Percentages From Maternal Iliofemoral Lymph Nodes at 20 dpi

**Figure 3:**
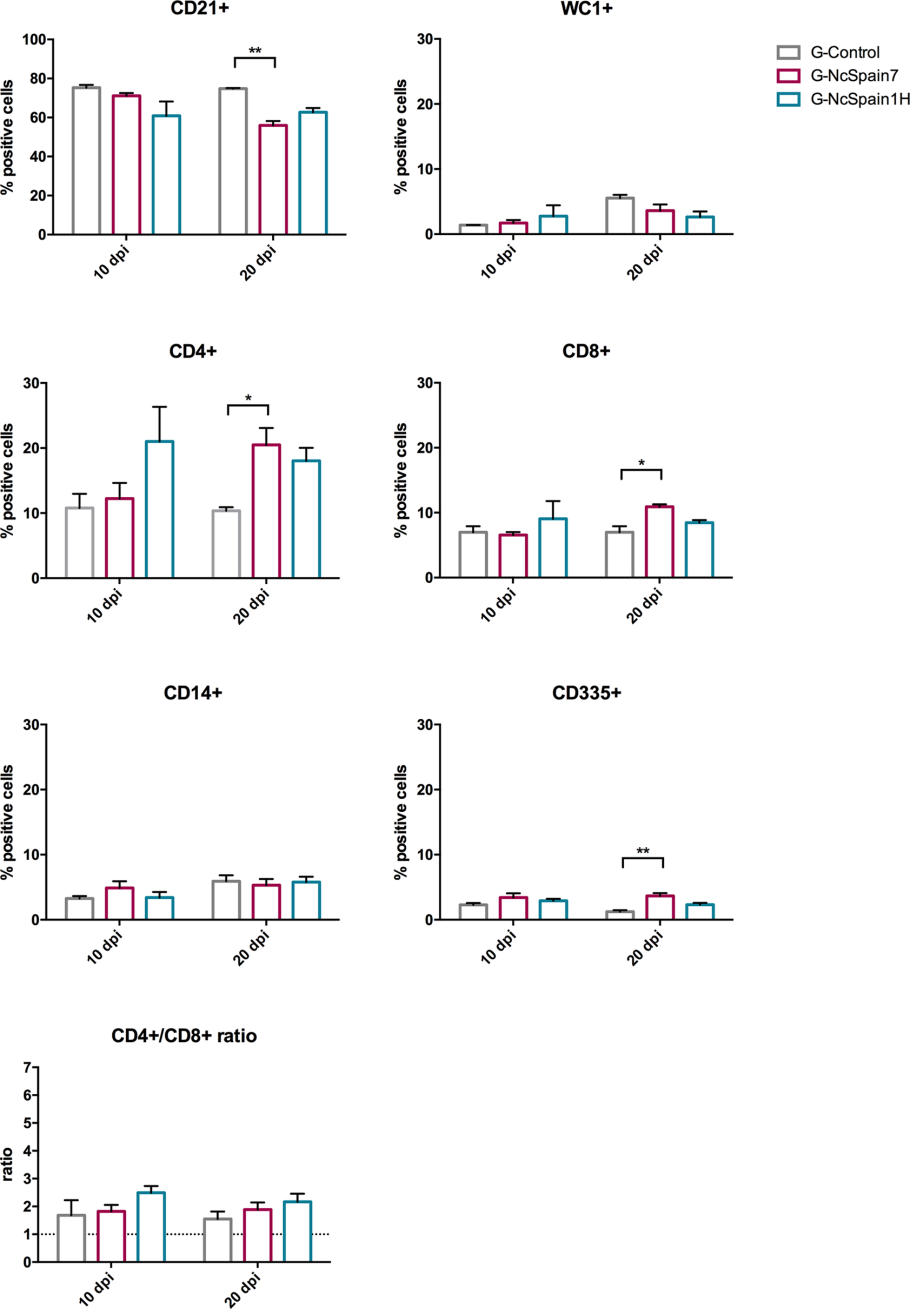
Relative percentage of immune cells in iliofemoral lymph nodes from Nc-Spain7- and Nc-Spain1H-infected heifers. Graphs indicate the relative percentage of cells positive for CD21, WC1, CD4, CD8, CD14 and CD335 surface markers, and CD4+/CD8+ ratio surface markers in the population of immune cells obtained from the iliofemoral lymph node of uninfected heifers (G-Control) and heifers challenged with Nc-Spain7 (G-NcSpain7) and Nc-Spain1H (G-NcSpain1H) tachyzoites culled at 10 or 20 dpi. The results are represented as the mean + SD. Asterisks indicate significant differences. **P < 0.01, *P < 0.05.

The same immune cell populations studied in PBMC were analysed in the iliofemoral lymph nodes of heifers infected with Nc-Spain7 and Nc-Spain1H and culled at 10 dpi or 20 dpi (**Figure 3**). Nc-Spain7-infected dams showed significantly lower percentages of CD21+ populations at 20 dpi (P<0.01), than uninfected dams. In addition, an increase in the CD4+ (P<0.05), CD8+ (P<0.05) and CD335+ (P<0.01) percentages in G-NcSpain7 *versus* G-control was found at 20 dpi. A higher CD4+ cell percentage was detected in G-NcSpain1H at 10 dpi, but the differences were not statistically significant due to a high variability observed between animals of this infection group. *Neospora caninum* infection had no impact on the percentage of CD14+, WC1+ cells or CD4+/CD8+ ratio for either of the isolates or time points assayed.

### Foetal Immune System Responded to Infection by *N. caninum* Isolates of High and Low Virulence

**Figure 4:**
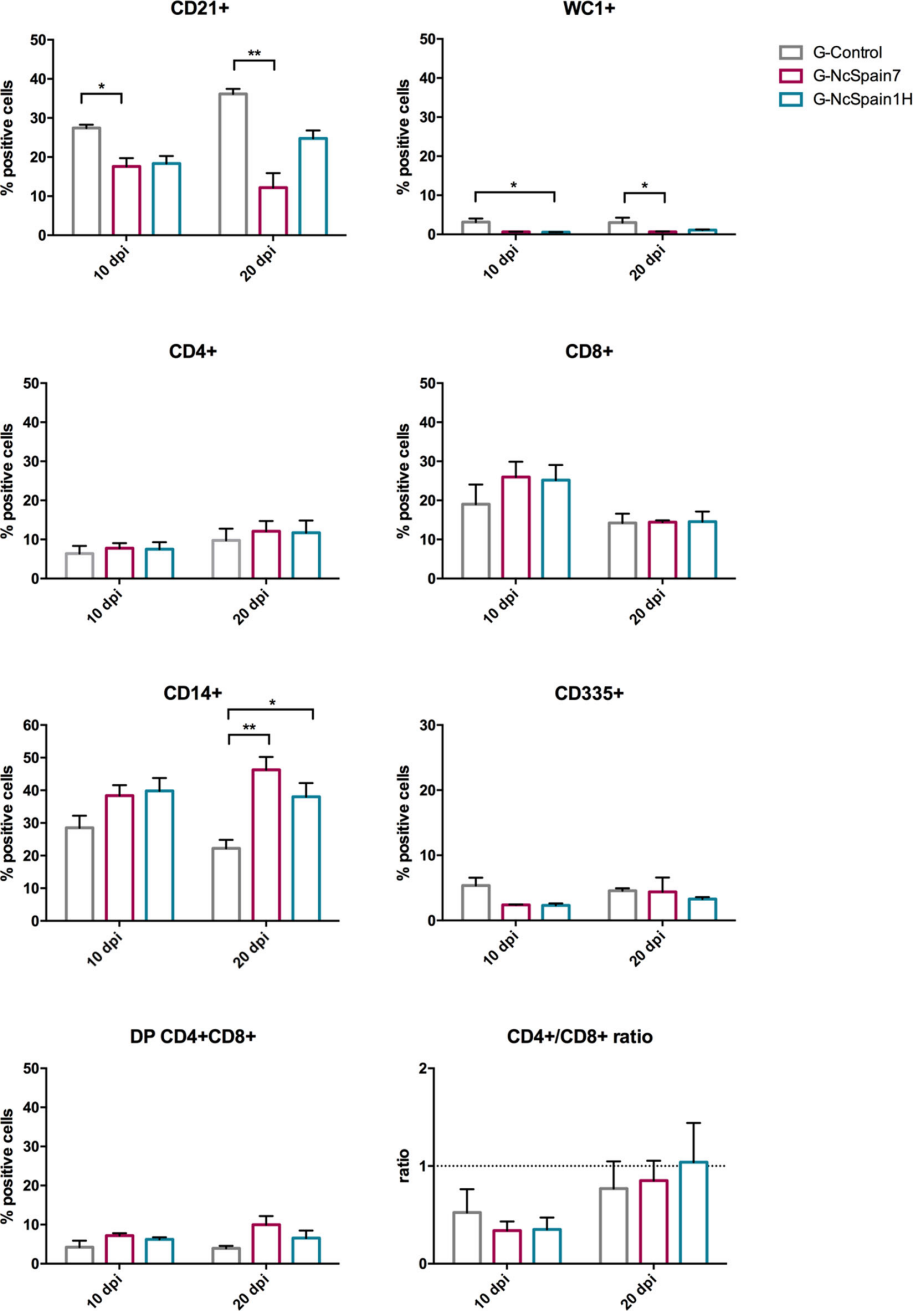
Immune cell populations in the spleen of foetuses from Nc-Spain7 and Nc-Spain1H infected heifers at 10 and 20 dpi. Graphs indicate the relative percentage of cells positive for CD21, WC1, CD4, CD8, CD14 and CD335 surface markers, double positive (DP) CD4+CD8+ cells and CD4+/CD8+ ratio, in the population of immune cells obtained from the spleen of foetuses from uninfected heifers (G-Control) and heifers challenged with Nc-Spain7 (G-NcSpain7) and Nc-Spain1H (G-NcSpain1H) tachyzoites culled at 10 or 20 dpi. The results are represented as the mean + SD. Asterisks indicate significant differences. **P < 0.01, *P < 0.05.

**Figure 5:**
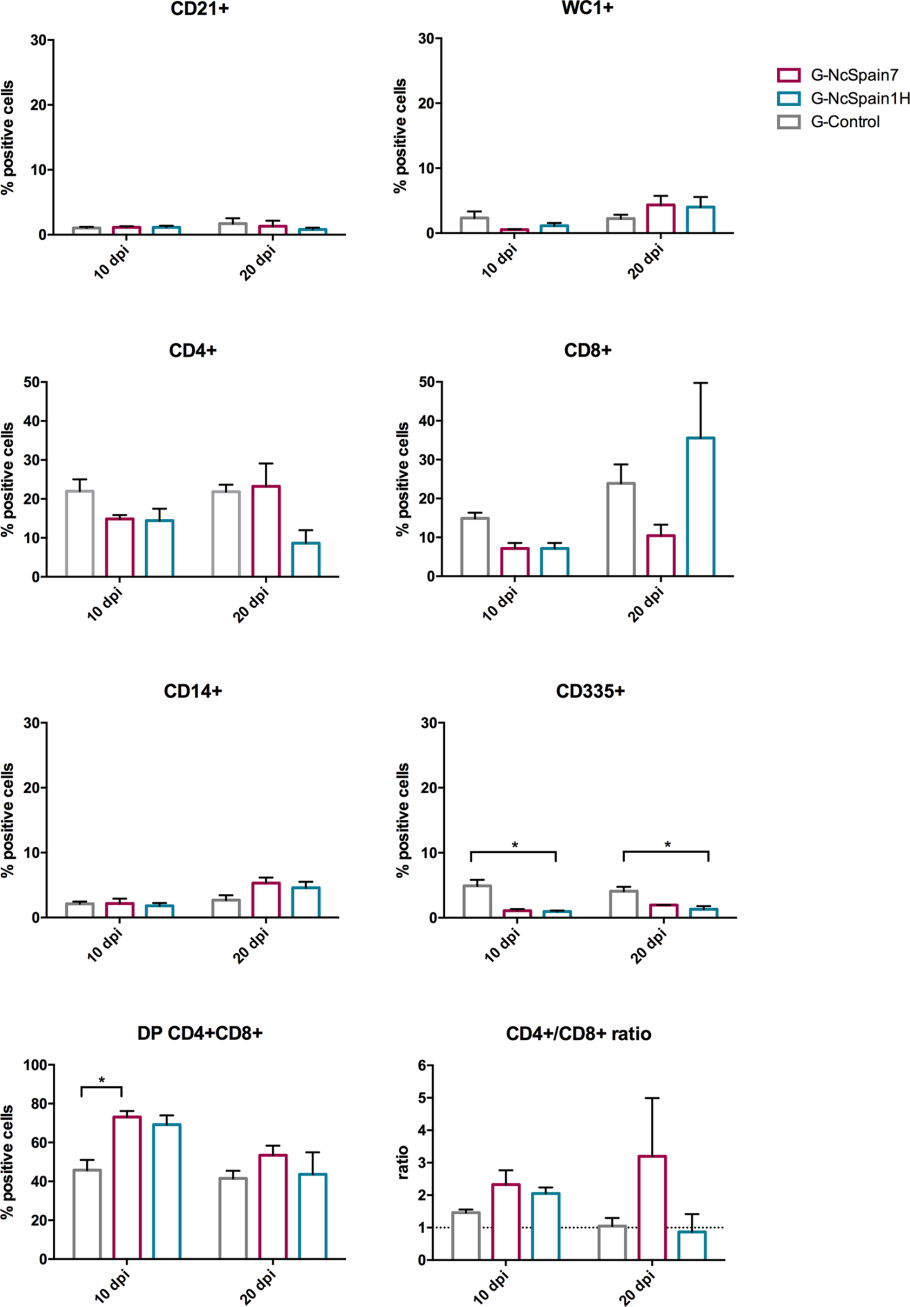
Immune cell populations in the thymus of foetuses from Nc-Spain7 and Nc-Spain1H infected heifers at 10 and 20 dpi. Graphs indicate the relative percentage of cells positive for CD21, WC1, CD4, CD8, CD14 and CD335 surface markers, double positive (DP) CD4+CD8+ cells and CD4+/CD8+ ratio, in the population of immune cells obtained from the thymus of foetuses from uninfected heifers (G-Control) and heifers challenged with Nc-Spain7 (G-NcSpain7) and Nc-Spain1H (G-NcSpain1H) tachyzoites culled at 10 or 20 dpi. The results are represented as the mean + SD. Asterisks indicate significant differences. *P < 0.05.

Foetal immune responses to *N. caninum* infection were studied by the analysis of immune cells in the spleen (**Figure 4**) and thymus (**Figure 5**). In both organs, we found cells double positive for CD4 and CD8 (up to 12% in the spleen and 70% in the thymus) in foetuses from infected and non-infected animals. In the foetal spleen, an increase in CD14+ cells in G-NcSpain7 (P < 0.01) and G-NcSpain1H (P < 0.05) was found at 20 dpi. In contrast, G-NcSpain7 induced a reduction in the percentage of CD21+ cells at 10 (P < 0.05) and 20 dpi (P < 0.01). In addition, the WC1+ cell percentage decreased at 10 dpi for G-NcSpain1H and at 20 dpi for G-NcSpain7 (P < 0.05). There were no changes in the CD4+, CD8+ and CD335+ *s*ubpopulation percentages or CD4+/CD8+ ratios.

In the thymus, an increase in the percentage of double CD4+CD8+ cells was observed for both isolates at 20 dpi, although this increase was only statistically significant for G-NcSpain7 (P < 0.05). In contrast, a reduction in the percentage of CD335+ cells in G-NcSpain1H foetuses at 10 and 20 dpi (P < 0.05) was observed.

## Discussion

In the present study, cellular immune response to *N. caninum* infection at mid gestation was studied with the aim of unravelling the immunological regulation that takes place at the initial events of infection (10 and 20 dpi) in pregnant cattle and their foetuses. We observed that independent of the isolate, dams mounted an early specific CMI response characterized mainly by an increase in the proportion of helper (CD4+) T cells at early infection (6 to 9 dpi) and cytotoxic (CD8+) T cells at late infection (9 to 20 dpi for Nc-Spain7 and 16 dpi for Nc-Spain1H). Previous studies also reported an increase in the percentages of CD4+ and CD8+ T cells in blood (Rosbottom et al., 2007) and lymph nodes (Almeria et al., 2003) after *Neospora* experimental infection in cattle. In addition it was demonstrated that T lymphocytes were able to recognize and respond to parasite-infected cells by producing IFN-γ, which is crucial for host defence against *N. caninum* (Innes et al., 2002; Innes et al., 2005; Williams et al., 2007). In the present study, following stimulation with *N. caninum* antigen, peripheral blood cells obtained from the infected cows were able to produce both IFN-γ and IL-4 from the end of the first week post-infection. In the same groups of animals as described here, we also reported a peak in IFN-γ levels in sera as early as 2 dpi in infected animals (Jiménez-Pelayo et al., 2019). According to our results, a very similar time course of cytokine production was observed previously (Rosbottom et al., 2007; Regidor-Cerrillo et al., 2014; Vázquez et al., 2019). In contrast, a decrease in the relative percentage of the B lymphocyte subset (CD21+) was observed in both infected groups. Previously, a decline in lymphocyte subpopulations after experimental *Neospora* infection in heifers at day 110 of gestation was also described (Almeria et al., 2003). Our finding may be a consequence of the increase in T cell populations, as an almost identical but inversely proportional profile was observed for CD21+ cells in relation to CD4+ T cells. Regarding γδ-T cells, previous *in vitro* studies have suggested that these cells may act as a short-term or innate response to *N. caninum* infection based on the capability of these cells to kill infected target cells upon cytokine stimulation (Peckham et al., 2014). Although *in vivo* studies have also demonstrated infiltration of γδ-T cells in placental tissues (Cantón et al., 2014; Hecker et al., 2015), our results suggest that γδ-T cells are not a main immune cell implicated in bovine peripheral immune responses against this parasite. In addition, NK cells did not experience any important changes after infection in our study. NK cells are important in innate defence against intracellular pathogens and act as early responders in *N. caninum* infections in bovine models (Klevar et al., 2007). Further investigations are necessary to investigate the potential role of γδ-T cells and NK cells in bovine neosporosis.

The ability of the foetal immune system to respond effectively to the parasite could be also of critical importance in determining the outcome of infection. However, the results from our study did not allow us to draw relevant conclusions, probably due to immaturity of the developing foetus at mid-gestation and the high variability between animals. Notably, a higher percentage of CD4+CD8+ double-positive cells was found in the thymus of *N. caninum-*infected fetuses at 10 dpi, indicating an active response against an ongoing infection. At 20 dpi, there was a reduction in the percentage of CD4+CD8+ double-positive cells in all the groups, possibly due to a higher level of maturation of the thymus at 130 days of gestation. In the spleen, the most remarkable result was an increase in the percentage of CD14+ cells at 20 dpi in fetuses from both infected groups. These findings again suggest that fetuses were able to respond to *N. caninum* infection at mid-gestation and that the spleen may have a greater responsiveness at this stage of pregnancy as has been previously suggested (Almeria et al., 2016).

When we compared the differential induction of maternal CMI response by infection with two *N. caninum* isolates showing marked differences in virulence traits (Jiménez-Pelayo et al., 2019; Jiménez-Pelayo et al., 2020), the results were rather similar but some differences were found. First, infection with the low-virulence Nc-Spain1H isolate induced an early increase in circulating CD14+ cells. Although individual differences were found, this result could be remarkable, since these cells play a main role in initiating early immune responses and priming the immune system for the development of adaptive immune responses against infection (Werling and Jungi, 2003; Jensen et al., 2018). A higher response of bovine macrophages to infection by Nc-Spain1H has been previously reported *in vitro* (García-Sánchez et al., 2019b; García-Sánchez et al., 2020) and was related to a higher expression of surface antigens (García-Sánchez et al., 2019a). Moreover, during the early response to an initial infection, mononuclear phagocytes are expected to encounter circulating parasites triggering an innate immune response (Goff et al., 2010). Consequently, phagocytic cells would reduce parasitaemia in the first multiplication cycles of the parasite, thereby possibly negatively affecting to parasite dissemination and the number of tachyzoites reaching the placenta. Second, an early CD4+ T cell increase was also induced by Nc-Spain1H infection in blood. CD4+ T cells could contribute to enhanced control of Nc-Spain1H growth and dissemination by releasing cytokines, activating mononuclear phagocytes, killing *N. caninum*-infected cells and affecting long-term disease progression. Consequently, a higher proportion of CD4+ lymphocytes is likely to be favourable. Previous reports have indicated that CD4+ T cells from experimentally infected cattle proliferate when stimulated with *N. caninum* antigen (Andrianarivo et al., 2001) and are able to inhibit tachyzoite multiplication by the resulting production of IFN-γ (Innes et al., 1995; Lundén et al., 1998; Rocchi et al., 2011). Here, although Nc-Spain1H infection induced lower IFN-γ levels than Nc-Spain7, the production was more maintained over time, and higher levels of IL-4 were induced by the infection with the low-virulent isolate. The ratio between helper and cytotoxic T cells was calculated as a marker of the immune regulation balance. This ratio could be used as a predictor of disease outcome. Various research studies have also described temporary changes in the CD4+/CD8+ ratio during some disease states in cattle (Ellis et al., 1990; Bassey and Collins, 1997; Chang et al., 2005). It is of interest that Nc-Spain1H triggered a shift in blood T lymphocyte subsets with a predominance of the CD4+ subpopulation, thereby raising the CD4+/CD8+ ratio. The role in pregnancy maintenance of a high CD4+/CD8+ was suggested previously (Hecker et al., 2013) and was associated with fewer foetal lesions, as we also observed in placental infiltration of animals infected with Nc-Spain1H (Jiménez-Pelayo et al., 2019; Jiménez-Pelayo et al., 2020). However, how a greater CD4+/CD8+ ratio may aid in protection in bovine neosporosis is unclear. Altogether, our results suggest that infection with the low-virulence isolate could be readily controlled by the developing immune response of dams and remarks the relevance of a balance of the immune response in the outcome of the infection. These results agree with our previous studies, in which the Nc-Spain1H isolate triggered early innate immune recognition in the bovine placenta, characterized by upregulation of genes involved in pathogen recognition inducing a solid Th1 response, which was counterbalanced by a higher expression of anti-inflammatory and regulatory cytokines, minimizing placental pathology and abortion (Jiménez-Pelayo et al., 2020).

In contrast, the highly virulent isolate Nc-Spain7 induced higher IFN-γ production by stimulated blood cells at the first week of infection, predominating over IL-4, which was observed previously and may be attributed to its higher proliferation capacity (Regidor-Cerrillo et al., 2014; Jiménez-Pelayo et al., 2017; García-Sánchez et al., 2019b). Although proinflammatory mediators may be beneficial to eliminate a high parasite load, overexpression may also cause immunopathology (Quin et al., 2002; Innes et al., 2007; Almería et al., 2017) compromising gestation, as we observed in the placentomes from these animals (Jiménez-Pelayo et al., 2020). High IFN-γ levels have been also associated with a failure in protection in different vaccine assays against neosporosis  (Vemulapalli et al., 2007; Aguado-Martínez et al., 2009). Thus, a Th1 immune response is not always associated with protection against the progression of *N. caninum* infection (but rather the contrary) and even significant levels of Th2 cytokines are important for controlling infection, especially during acute infection  (Nishikawa et al., 2001; Haldorson et al., 2005).

However, the largest differences between isolates were observed in CD8+ cells in PBMC, which appeared in a significantly higher proportion from 9 dpi onwards. CD8 is a marker for cytotoxic T cells, which are largely involved in the recognition and killing of cells infected with intracellular pathogens. Staska et al. (2003) showed that CD8+ cytotoxic lymphocytes from cattle could kill *N. caninum*-infected autologous target cells *in vitro*. The CD8+ increase would be an attempt by the dam to reduce parasite multiplication mediated directly by lysis of infected cells as a consequence of the higher invasion and replication rates of Nc-Spain7 (Regidor-Cerrillo et al., 2011; Jiménez-Pelayo et al., 2017; García-Sánchez et al., 2019b). However, CD8+ increases could enhance tissue damage *in vivo* due to cytotoxic effects (Correia et al., 2015). Previously, we described that CD8 + lymphocytes predominated in placentomes from G-Nc-Spain7 and that severe placental damage and foetal death were found (Jiménez-Pelayo et al., 2020). In this respect, Cantón et al. (2014) also suggested a positive association between a high number of CD8+ T lymphocytes in the placental infiltrate and the occurrence of abortion. Although speculative, an alternative interpretation for this increase in CD8+ cells could also be related to immune evasion mechanisms triggered by the high-virulence isolate, since CD8+ lymphocytes can also exert suppressor effector functions (Inoue et al., 1993; Rocamora-Reverte et al., 2020). Thus, an increase in the percentage of CD8+ lymphocytes may suppress the activity of CD4+ T lymphocytes (Shafer-Weaver and Sordillo, 1997). Nc-Spain7 infection also induced a delayed increase in the percentage of monocytes/macrophages in blood and CD4+ T lymphocytes compared with Nc-Spain1H, which could contribute to delayed host immune responsiveness during the early stages of pathogenesis. In line with these results, we have more evidence supporting that Nc-Spain7 elicits mechanisms to circumvent the immune response that could allow survival during early infection (García-Sánchez et al., 2019a; García-Sánchez et al., 2019b; García-Sánchez et al., 2020; Jiménez-Pelayo et al., 2020). In addition, only Nc-Spain7 induced changes in the immune cell population at 20 dpi from the maternal iliofemoral lymph node, in which the uterus drains. This result could be clinically correlated with the presence of parasites in the placenta and foetus of Nc-Spain7-infected groups and the occurrence of abortion (Jiménez-Pelayo et al., 2019).

## Conclusions

This study shows that isolates of different virulence may have an inherent ability to trigger different host cellular immune responses in a pregnant cow that these abilities vary in the initial recognition of the pathogen, and the character and duration of the subsequent response. Our results support the hypothesis put forward in previous *in vivo* and *in vitro* studies suggesting that the high-virulence isolate Nc-Spain7 may induce a delayed activation of CD14+ cells. This would favour its survival and arrival at the placenta, where due to its greater proliferative capacity, it would cause tissue damage enhanced by a predominant Th1 immune response. In contrast, early recognition of the low-virulence isolate Nc-Spain1H and the induction of robust responses with a better immunoregulatory balance, although not eliminating the infection, would reduce parasitemia and thus the number of parasites reaching the placenta, allowing foetal survival. We also suggested that some potential immune traits, such as IFN-γ and IL-4 levels, as well as the CD4+/CD8+ ratio and could be correlated with protection against abortion (higher IL-4 levels and CD4+/CD8+ ratio) or with poor prognosis or inflammatory conditions (higher IFN-γ production and increase of CD8+ lymphocytes).

## Data Availability Statement

The raw data supporting the conclusions of this article will be made available by the authors, without undue reservation.

## Ethics Statement

The animal study was reviewed and approved by Animal Research Ethics (reference number PROAE 25/2016), following the proceedings described in Spanish and EU legislations (Law 32/2007, R.D. 53/2013, and Council Directive 2010/63/EU).

## Author Contributions

JR-C, PH, KO, LO-M, and EC-F conceived the study and participated in its design. MG-S and EC-F wrote the manuscript, with interpretation of results and discussion inputs from JR-C, PH, and LO-M. PH prepared the parasite inocula. MG-S, LJ-P, PV, and AJ-M performed the collection of the samples. MG-S and LJ-P analysed the samples by flow cytometry and ELISA, carried out statistical analyses and interpreted the results. All authors contributed to the article and approved the submitted version.

## Funding

This work was supported by the Spanish Ministry of Economy and Competitiveness (AGL2013-44694-R and AGL2016-75935-C2-1-R) and the Community of Madrid (PLATESA2-CM P2018/BAA-4370). MG-S was financially supported through a grant from the Spanish Ministry of Economy and Competitiveness (BES-2014-070723) and LJ-P was financially supported by a fellowship from the University Complutense of Madrid.

## Conflict of Interest

The authors declare that the research was conducted in the absence of any commercial or financial relationships that could be construed as a potential conflict of interest.
