# Supplementary material for: Maternal and Foetal Cellular Immune Responses in Dams Infected With High- and Low- Virulence Isolates of Neospora caninum at Mid-Gestation
Source: Front Cell Infect Microbiol. 2021 Jun 22;11:684670. doi: 10.3389/fcimb.2021.684670 (PMC8259741; doi:10.3389/fcimb.2021.684670)
Supplement: Supplementary file 1 [file DataSheet_1.pdf]

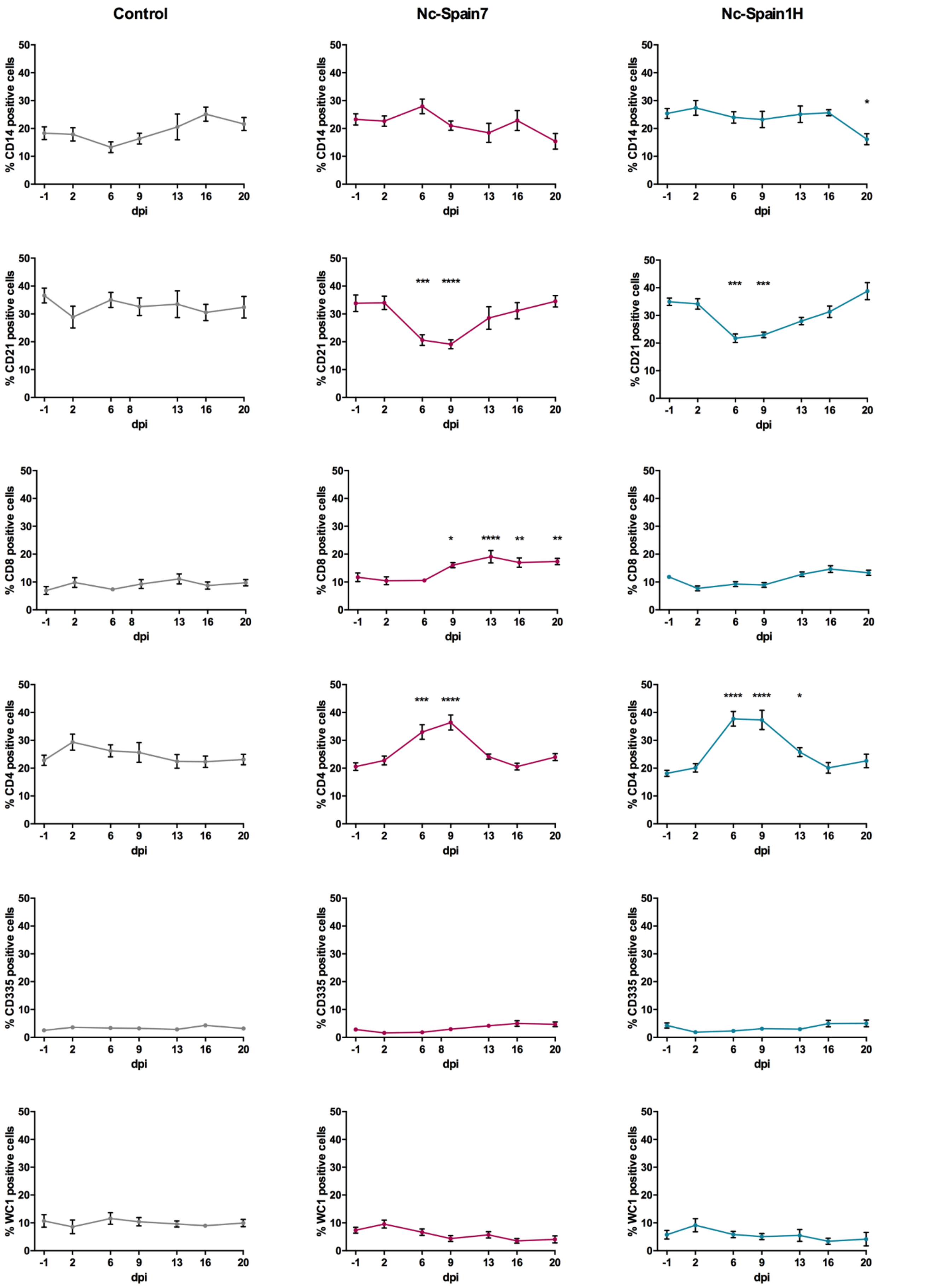

Supplementary S1. Relative percentage of immune cells in a PBMC population from Nc-Spain7 and Nc-Spain1H infected heifers. Graphs indicate the relative percentage of cells positive for CD21, WC1, CD4, CD8, CD14 and CD335 surface markers in PBMC obtained from uninfected heifers (G-Control) and heifers challenged with Nc-Spain7 (G-NcSpain7) and Nc-Spain1H (G-NcSpain1H) tachyzoites in relation to pre-infection values (-1 dpi). Asterisks indicate significant differences. ****$P < 0.0001$, ***$P < 0.001$, **$P < 0.01$, *$P < 0.05$.
